# RELATIONSHIPS BETWEEN DISTRESS AND NEIGHBORHOOD CHARACTERISTICS FOR MEXICAN AMERICAN CAREGIVERS

**DOI:** 10.1093/geroni/igad104.3564

**Published:** 2023-12-21

**Authors:** Florence Johnson, Stipica Mudrazija, Sheria Robinson-Lane, Shekinah Fashaw-Walters, Phillip Cantu

**Affiliations:** University of Michigan, Ann Arbor, Michigan, United States; University of Washington, Seattle, Washington, United States; University of Michigan School of Nursing, Ann Arbor, Michigan, United States; University of Minnesota, Minneapolis, Minnesota, United States; UTMB, Galveston, Texas, United States

## Abstract

Previous study demonstrates that caring for older relatives with physical or cognitive disability can increase caregiver stress—particularly if neuropsychiatric symptoms are present. The literature suggests that community features such as the percentage of immigrants may improve Mexican-American caregivers’ mental health. Using matched 2011/2012 data on caregivers and care recipients from the Hispanic Established Population for the Epidemiological Study of the Elderly(H-EPESE) and the 2010 census tract characteristics from the National Neighborhood Data Archive (NANDA) recently, we fit multilevel regression models to assess how neighborhood characteristics (census tract proportion Latino, proportion foreign-born, and proportion in poverty) affect caregiver distress (caregiver depression score, caregiver general stress, caregiver reports of neuropsychiatric behavioral symptoms of cognitive decline and caregiver report of stress from neuropsychiatric behavioral symptoms of cognitive decline). We found that living in an area with a higher proportion of foreign-born adults is related to lower reports of neuropsychiatric symptoms and lower levels of caregiver distress. Family caregivers residing in ethnic enclaves with high proportions of foreign-born persons reported significantly fewer symptoms of feeling stubborn and resistive, being in low spirits, feeling upset when separated from the care recipient, losing interest in usual activities, or experiencing changes in weight. While residing in these communities may reduce behavioral concerns in care recipients and increase social support availability, cultural factors such as ideas about normative aging may influence symptom reporting. Increasing available support for caregivers residing in ethnic enclaves is an important step toward developing dementia-capable communities and reducing caregiver health disparities.

